# Transcriptome analysis of the transdifferentiation of canine BMSCs into insulin producing cells

**DOI:** 10.1186/s12864-021-07426-3

**Published:** 2021-02-25

**Authors:** Jinglu Wang, Pengxiu Dai, Tong Zou, Yangou Lv, Wen Zhao, Xinke Zhang, Yihua Zhang

**Affiliations:** The College of Veterinary Medicine of the Northwest Agriculture and Forestry University, No.3 Taicheng Road, Yangling, 712100 Shaanxi P. R. China

**Keywords:** BMSC, Sequencing, Insulin producing cells, Transdifferentiation, PPI, *VIP*, *SSTR2*, *RPS6KA6*

## Abstract

**Background:**

Bone marrow mesenchymal stem cells are a potential resource for the clinical therapy of certain diseases. Canine, as a companion animal, living in the same space with human, is an ideal new model for human diseases research. Because of the high prevalence of diabetes, alternative transplantation islets resource (i.e. insulin producing cells) for diabetes treatment will be in urgent need, which makes our research on the transdifferentiation of Bone marrow mesenchymal stem cells into insulin producing cells become more important.

**Result:**

In this study, we completed the transdifferentiation process and achieved the transcriptome profiling of five samples with two biological duplicates, namely, “BMSCs”, “islets”, “stage 1”, “stage 2” and “stage 3”, and the latter three samples were achieved on the second, fifth and eighth day of induction. A total of 11,530 differentially expressed transcripts were revealed in the profiling data. The enrichment analysis of differentially expressed genes revealed several signaling pathways that are essential for regulating proliferation and transdifferentiation, including focal adhesion, ECM-receptor interaction, tight junction, protein digestion and absorption, and the Rap1 signaling pathway. Meanwhile, the obtained protein–protein interaction network and functional identification indicating involvement of three genes, *SSTR2*, *RPS6KA6*, and *VIP* could act as a foundation for further research.

**Conclusion:**

In conclusion, to the best of our knowledge, this is the first survey of the transdifferentiation of canine BMSCs into insulin-producing cells according with the timeline using next-generation sequencing technology. The three key genes we pick out may regulate decisive genes during the development of transdifferentiation of insulin producing cells.

**Supplementary Information:**

The online version contains supplementary material available at 10.1186/s12864-021-07426-3.

## Background

Stem cells in particular for BMSCs have been used for decades for the treatment of many diseases. Numerous reports about the therapeutic potential of BMSCs have been published. BMSCs can be used for the regeneration of cartilage and osteochondral tissue defects [1], craniofacial tissue [2], and spinal cord [3]; moreover, type 1 diabetes can be treated using BMSC-derived insulin-producing cells [4]. The number of patients with diabetes is continuing to increase; according to the WHO (https://www.who.int/diabetes/global-report/en/), in 2016, 422 million individuals were affected by diabetes globally [5].

Pancreatic islet transplantation is available as an alternative therapy for diabetes, but it has the limitation of insufficient availability of islets; as such, many research teams have searched for other cells that could substitute for islets. Human embryonic stem cell (hESC)- [6] and induced pluripotent stem cell (iPSC)-derived islet-like cells [7] have primarily been used to form islet-like clusters, but this is associated with a relatively high risk of neoplasia [8, 9] and other ethical issues [10]. Against this background, induced β cells derived from BMSCs are a promising option given that they are easy to obtain and immunoregulation [11, 12], and they can differentiate into osteoblasts, chondrocytes, and adipocytes [13, 14] in vitro. There are many ways to obtain insulin-producing cells using BMSCs. For example, it is possible to directly convert BMSCs into β-like cells by the lentiviral transduction of *NGN3*, *PDX1*, and *MAFA* [15]. Moreover, the addition of pancreas extract to the culture medium can be effective [16]. Another option is reprogramming, which can be achieved via the supplementation of small molecules such as conophylline [17].

Other groups reported that BMSC-derived vascular endothelial growth factor (VEGF) [18], epidermal growth factor (EGF) [19], and insulin-like growth factor 1 (IGF-1) [20] exhibited protective effects in many disease models and that the overexpression of several factors could indirectly mediate tissue repair [21]. The procedure for inducing pancreatic islet-like cells that we used in this study requires various factors, including pathway inhibitors and components EGF, bFGF, activin A, exendin-4, betacellulin, and nicotinamide [22–27], and it was a high efficient process compared with other research [26]. With this induction procedure, over 60 percentage human BMSCs turned into islet-like cell clusters [26, 27].

Different signaling pathways are involved when reprogramming occurs. For example, the AKT signaling pathway influences hypoxic stress and STZ stimulation [28], while ERK1/2 signaling pathway regulation confers resistance to apoptosis [29]. Many animal experiments have shown that co-transplantation of pancreatic islets and BMSCs can boost the survival rate of islets, which improves the efficiency of surgery [30]. Research has also demonstrated the essential role played by the extracellular matrix, directly interacting with cells, in determining the direction and fate of cell differentiation [31]. In summary, signaling pathways, the extracellular matrix, and transplantation methods can influence the rate of islet-like cell mass acquisition and the therapeutic efficiency of transplantation.

However, all of the available reprogramming methods are limited by the low efficiency of transformation [26, 32–34], which may lead to an insufficiency of pancreatic islets for transplantation surgery. This problem is associated with our limited understanding of the transdifferentiation mechanisms.

In this study, BMSCs were converted to islet-like cells by a three-step induction procedure and samples were collected at the end of each phase along with BMSCs and islets as negative and positive controls. Then, they were analyzed by transcriptome analysis, followed by enrichment analysis and PPI network analysis with three genes chosen for further research. The results obtained here could provide a foundation for future work to understand the mechanisms underlying the transdifferentiation of canine BMSCs into islet-like structures.

## Results

### Isolation and identification of BMSCs

**Fig. 1:**
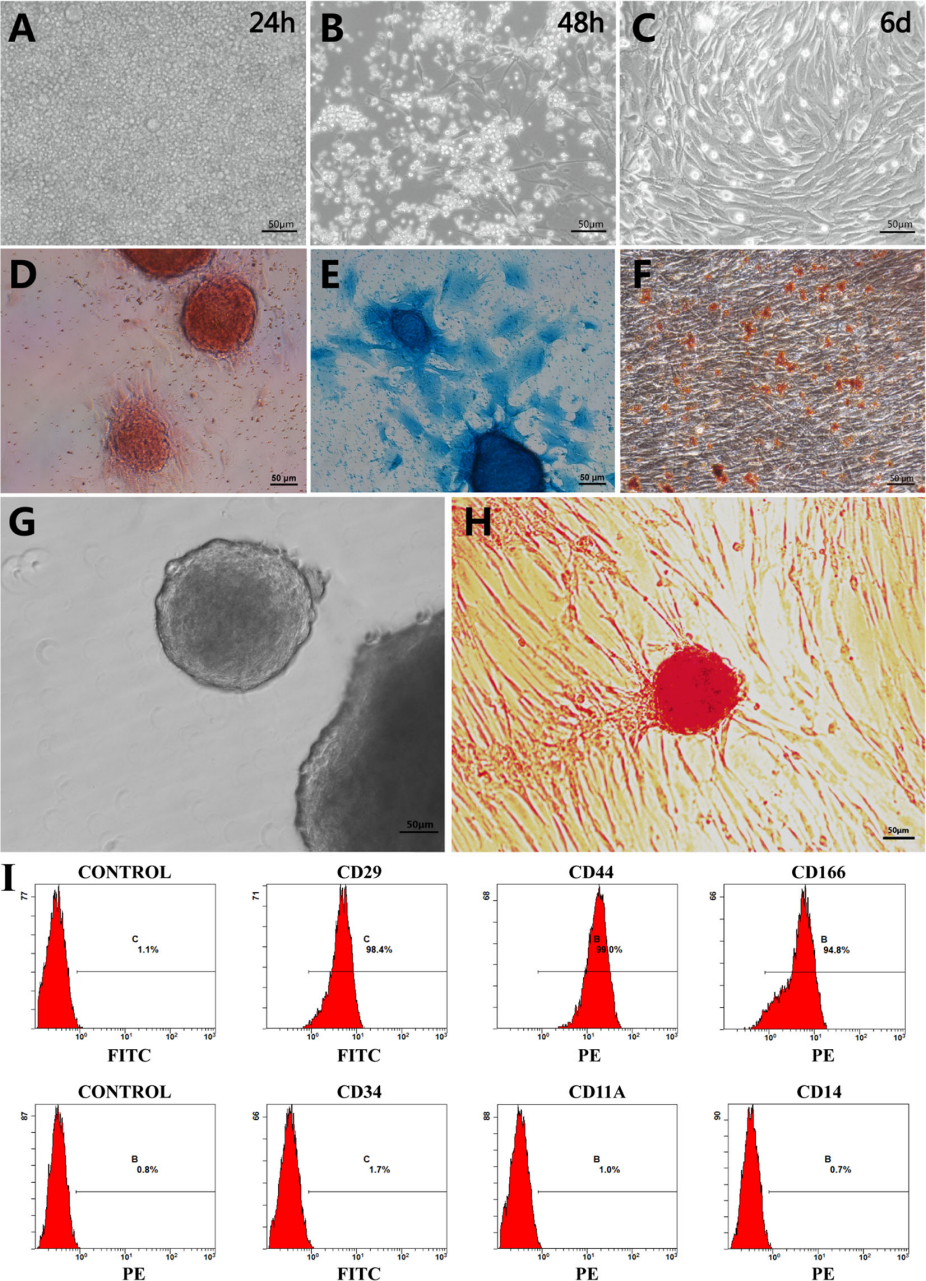
The separation, identification and induction of BMSCs. **a** 24 h after the separation, only blood cells could be seen in the dish. **b** Two days later, with replacement of medium several attached BMSCs, which were in spindle shape, could be seen. **c** When it came to 6 days, the BMSCs grew vortically. **d** This was the alizarin red staining for the osteoblasts, which meant that BMSCs could different into osteoblasts. **e** The differentiation of BMSCs to chondroblasts was confirmed by alcian blue staining. **f** BMSCs were positive for the oil red O staining when BMSCs were induced to adipocytes. **g** The induction procedure was performed under a nonadherent state and the spheroids were ranged from 100 to 200 μm. **h** After the last step of induction, the reattached clusters were stained by DTZ, and they showed brownish red. (scale bar = 50 μm). **i** The flow cytometry of BMSCs

When we transferred the mixture of cells extracted from bone marrow into dishes, we could only see the blood cells floating around, which is a necessary initial niche for the development of BMSCs. After 24 h of culture, the canine BMSCs presented an adherent state and fiber-like or irregular morphology; they also turned into spiral clusters when approaching confluence, which took approximately 6 days (Fig. 1a-c).

The results of flow cytometry assay confirmed that the BMSCs were positive for CD29, CD44, and CD166, and negative for CD11a, CD14, and CD34 (Fig. 1i).

To confirm the differentiation ability of BMSCs, we performed a three-lineage differentiation experiment; here, cell status was determined by staining tests, namely, Alizarin Red for osteoblasts (Fig. 1d), Alcian Blue for chondroblasts (Fig. 1e), and Oil Red O for adipocytes (Fig. 1f).

### Induction and characterization of islet-like spheroids

At the nonadherent stage, BMSCs formed many spheroids floating in the medium (Fig. 1g). After the last induction stage, the spheroids were all positive for DTZ staining (Fig. 1h). The results of GSIS, 8.76 μIU/mL for DMEM low glucose (10 mM) and 45.22 μIU/mL for DMEM high glucose (25 mM) (Fig. 7ki), confirmed that BMSCs had been transformed to pancreatic islet-like state.

### De novo assembly

**Fig. 2:**
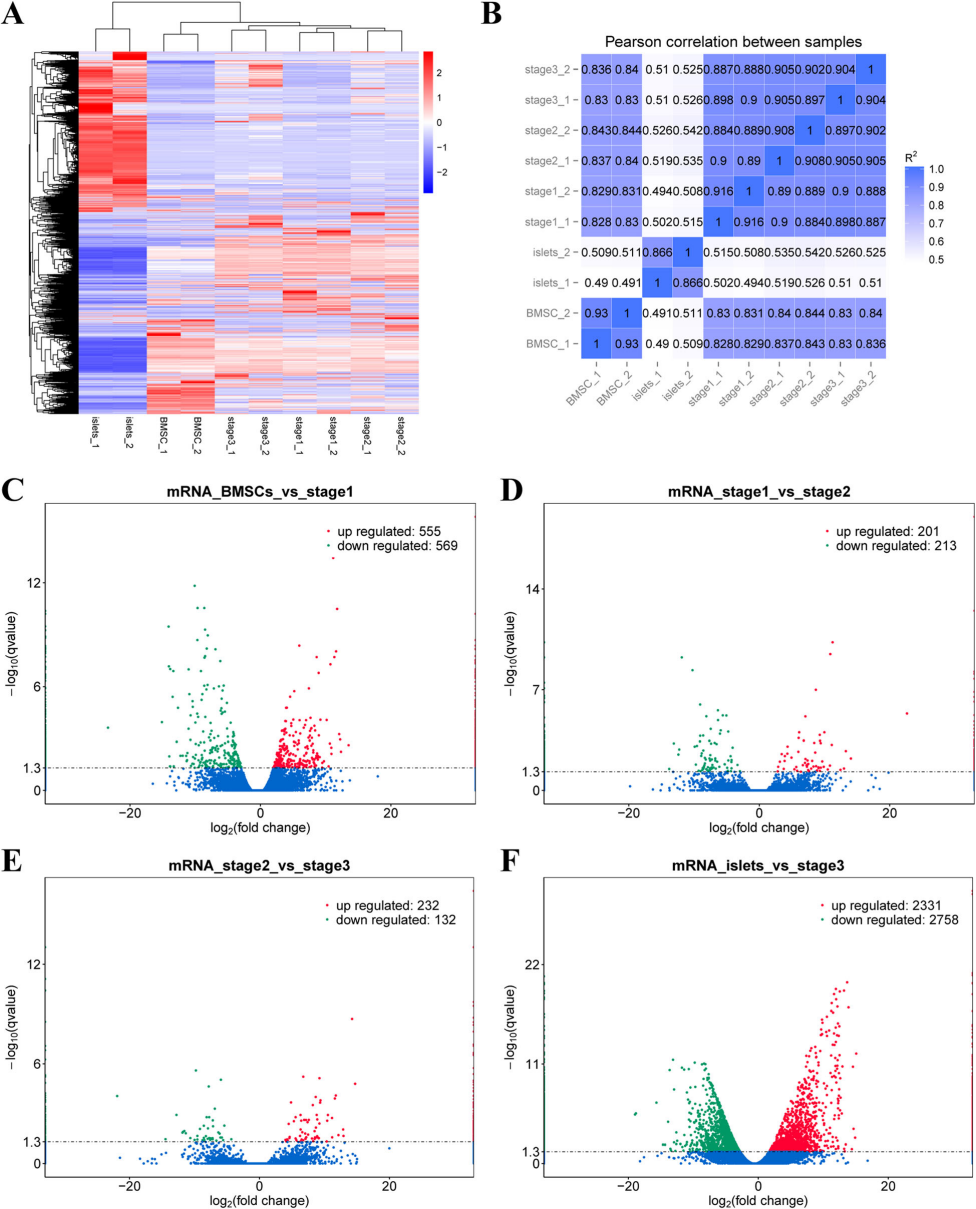
DEGs of different groups. **a** Heatmap illustrated differentially expressed genes of known transcripts which were screened based on |log2FC| > 1 and FDR < 0.05. **b** The person correlation coefficients of every two samples and the repeatability of these samples were confirmed, meanwhile, the variation of different samples could also be seen. **c**-**f** The volcano maps for up regulated and down regulated genes between different samples “BMSCs vs stage1”, “stage1 vs stage2”, “stage2 vs stage3” and “islets vs stage3”

Pearson’s correlation coefficients (R^2^) were all around 0.9 for each biological replication of samples, which meant that these data were repeatable (Fig. 2b). The raw reads for each sample numbered around 100 million and the clean reads numbered about 90 million. Q20 and Q30 ranged from 96.67 to 97.12% and 91.87 to 92.76%, respectively, while the GC content ranged from 44.83 to 56.19% (Additional file 1). All clean reads, filtered by HISAT2, were used for comparison with the reference genome; in order for the reference genome to be considered suitable for this analysis and samples not to be contaminated, the rate of mapped reads should exceed 70%. The rate of total mapped reads was over 90% and the rates of uniquely mapped reads were all above 80% as prediction. (Additional file 2).

The quantitative analysis of common types of genes, such as miRNAs, tRNAs, and SRP-RNAs, using the software HTSeq, provided information on the status of different gene types based on the gene expression volume. The majority of genes were “protein-coding genes,” representing 67.60% of the total on average.

### Overview of DEGs

Based on the thresholds of |log2FC| > 1 and FDR < 0.05, we subjected all types of transcripts to differential expression analysis. Differential transcript cluster analysis of mRNAs showed that there were 11,530 differentially expressed transcripts (Fig. 2a). The comparison of “BMSCs” and “stage1” showed that there were 555 upregulated genes and 569 downregulated ones (Fig. 2c), while there were 201 upregulated genes and 213 downregulated ones for the comparison between “stage 1” and “stage 2” (Fig. 2d), 232 and 132 for “stage 2” and “stage 3” (Fig. 2e), and 2331 and 2758 for “islets” vs “stage 3,” respectively (Fig. 2f).

### Enrichment analysis of DEGs from transcriptome sequencing and GEO data

**Fig. 3:**
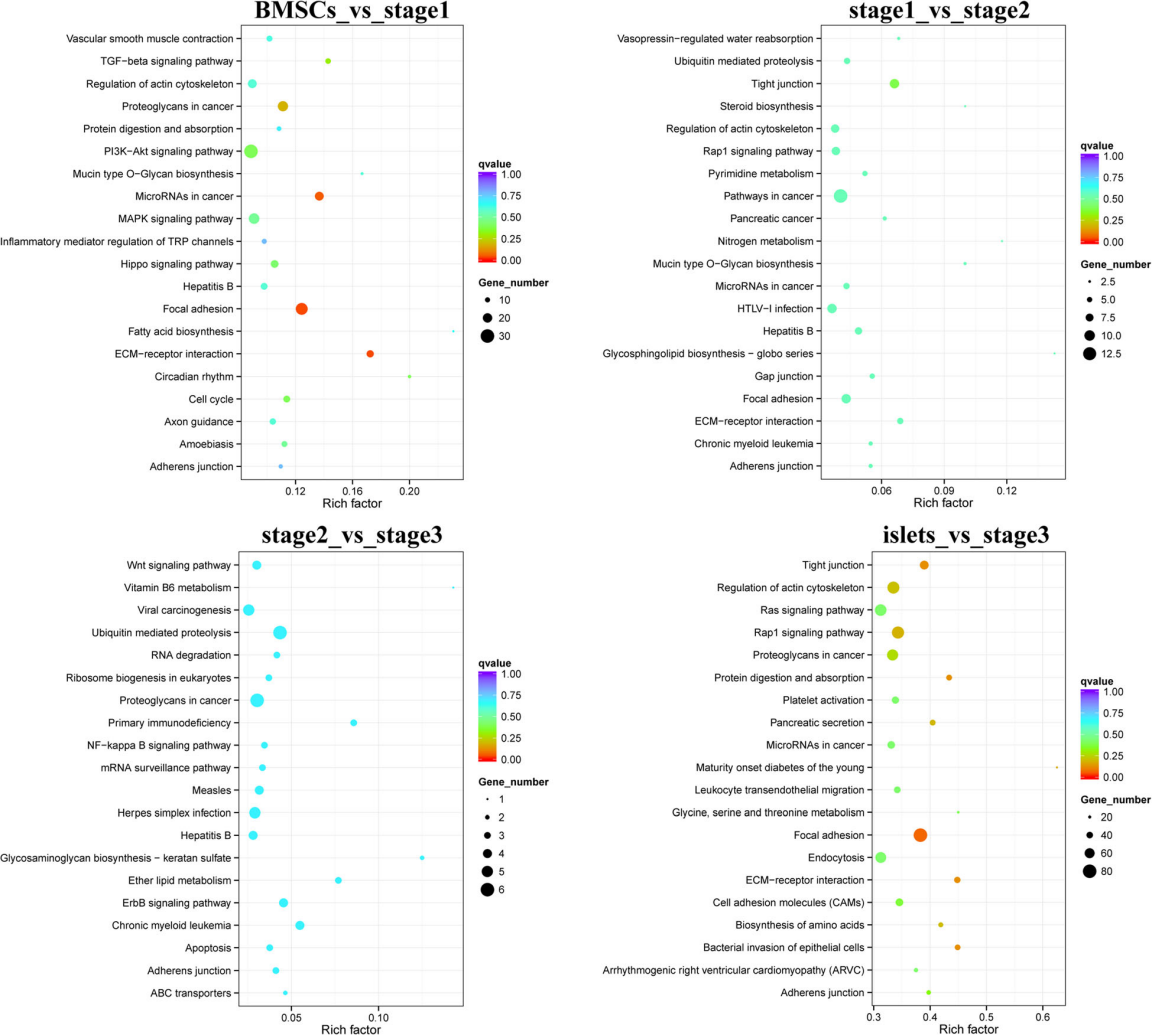
Top 20 KEGG signaling pathways of known transcripts, the size of bubbles represented for mapped gene numbers of the item, and the color was measured by the Qvalue. **a** KEGG analysis of “BMSCs vs stage 1”. **b** KEGG analysis of “stage 1 vs stage 2”. **c** KEGG analysis of “stage 2 vs stage 3”. **d** KEGG analysis of “islets vs stage 3”

All of the DEGs, including upregulated and downregulated transcripts, were annotated to signaling pathways related to the GO terms. For the KEGG pathway analysis, the “BMSCs vs. stage 1” DEGs were mapped to 230 KEGG pathways, while the “stage 1 vs. stage 2,” “stage 2 vs. stage 3,” and “islets vs. stage 3” DEGs were annotated to 163, 148, and 273 pathways, respectively. Considering RF and Q-value, we obtained the top 20 KEGG pathways for each comparison group, and they shared several of the same pathways, including tight junction, protein digestion and absorption, pancreatic secretion, focal adhesion, ECM-receptor interaction, Rap1 signaling pathway, and cell cycle (Fig. 3a-d). These pathways are significantly related to cell proliferation and differentiation, which control the fate of BMSCs when induce to differentiate.

**Fig. 4:**
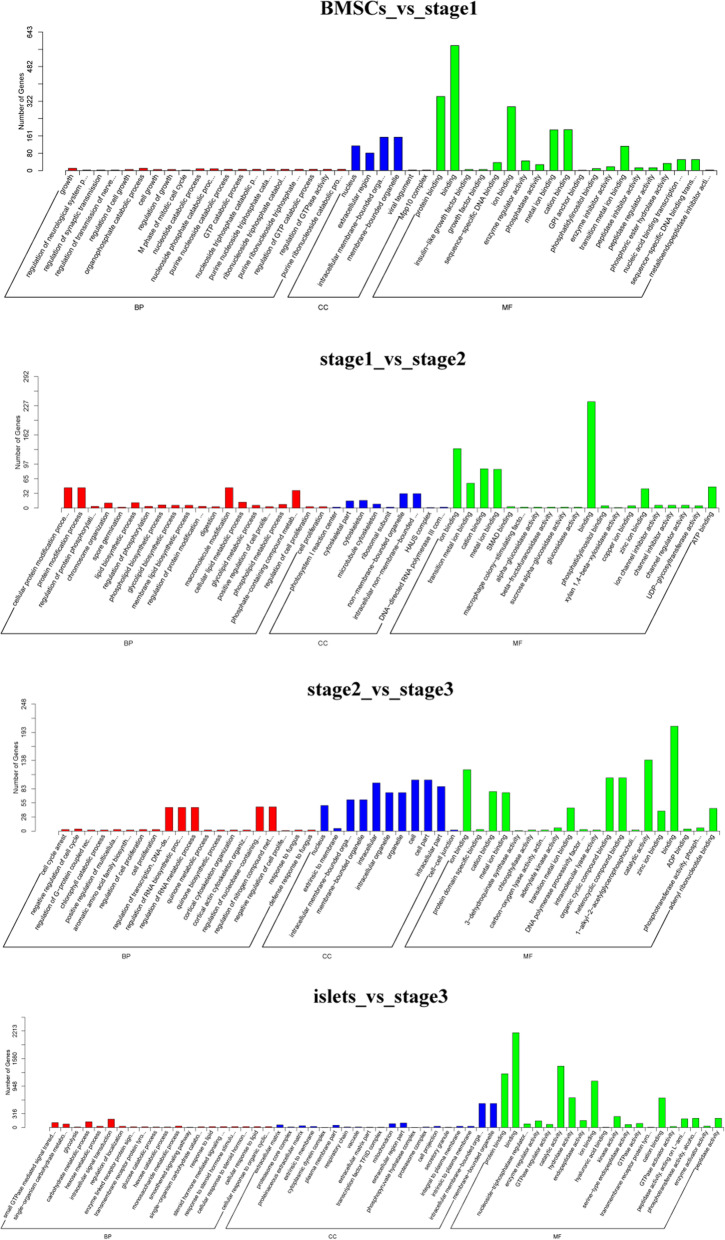
Top 20 GO items demonstrated that the MF and CC usually are the dominate part of GO. **a** GO analysis for “BMSCs vs stage1”. **b** GO analysis for “stage1 vs stage2”. **c** GO analysis for “stage2 vs stage3”. **d** GO analysis for “islets vs stage3”

The GO analysis of “BMSCs vs. stage 1” was dominated by CC and MF, which included the categories of nucleus, extracellular region, intracellular membrane-bound organelle, membrane-bound organelle; and protein binding, binding, ion binding, metal ion binding, cation binding, and transition metal ion binding (Fig. 4a). For “stage 1 vs. stage 2,” the DEGs were mainly annotated to the categories of cellular protein modification process, protein modification process, and phosphate-containing compound metabolic process for BP; cytoskeleton, non-membrane-bound organelle, and intracellular non-membrane-bound organelle for CC; and ion binding, cation binding, metal ion binding, binding, zinc ion binding, and ATP binding for MF (Fig. 4b). The comparison between “stage 2” and “stage 3” showed that binding activity still formed the majority of MF and that CC was still dominated by organelle; however, for BP, the main categories were the regulation of transcription, regulation of RNA biosynthetic process, and regulation of RNA metabolic process (Fig. 4c). Then, for the pair “islets vs. stage 3,” annotations included binding activities like those mentioned above for MF; additional extracellular region part, extracellular matrix, and mitochondrion for CC; and intracellular signal transduction, carbohydrate metabolic process, single-organism carbohydrate metabolic process, and small GTPase-mediated signal transduction for BP (Fig. 4d).

**Fig. 5:**
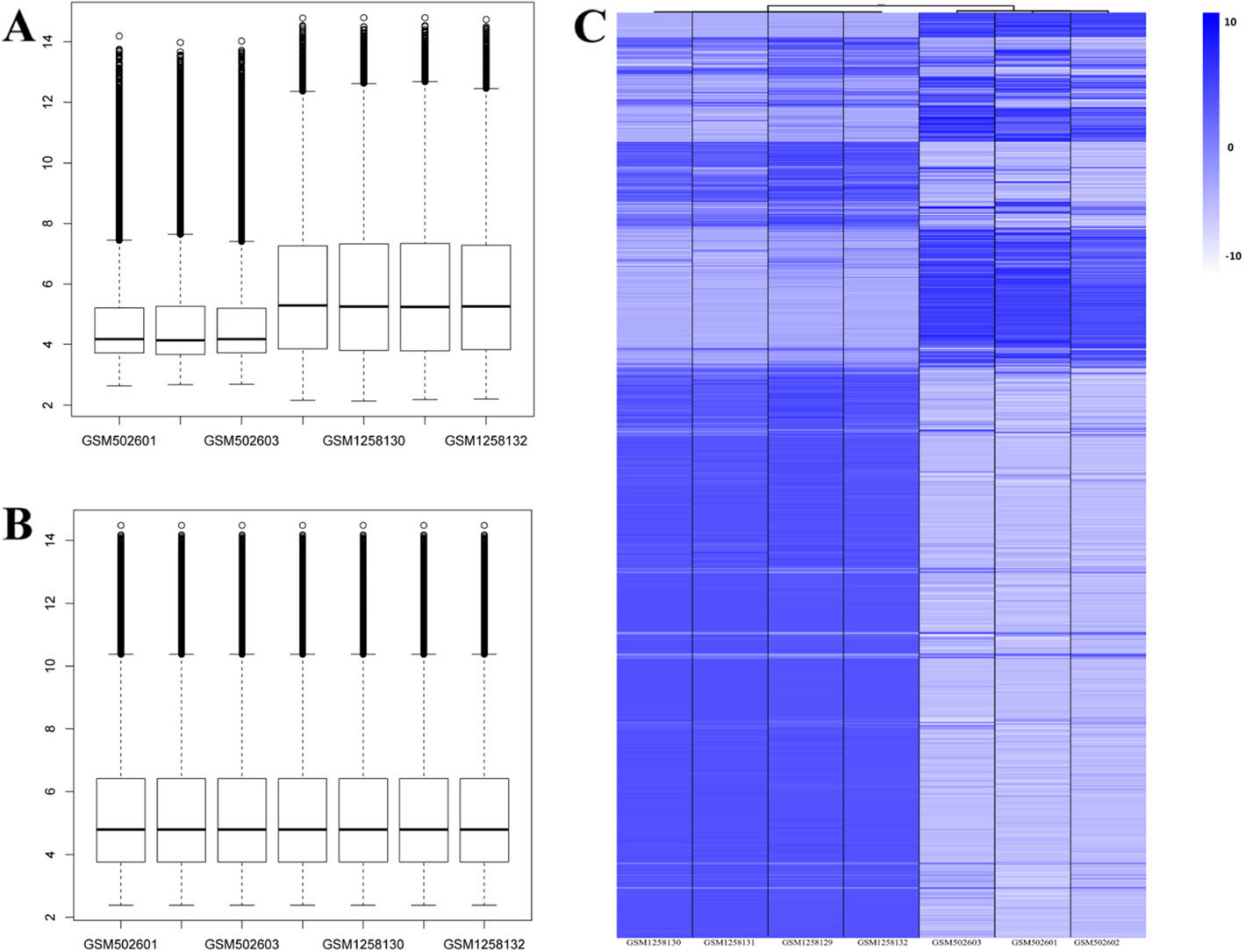
Normalization and enrichment analysis of GEO data. **a** The status of the raw data from GEO database showed in box-plot. **b** Normalized data showed in box-plot, and the median was at the same level. **c** DEGs of GEO data, the expression level low to high were showed by light to dark blue

To further verify the results, we downloaded similar sample sources from GEO database for the comparison with our results. They were chosen from two datasets, GSE20113 and GSE52063, which belong to the same platform, GPL3738. We also chose three normal pancreas samples, namely, GSM502601, GSM502602, and GSM502603, from the first dataset, along with four BMSC samples, namely, GSM1258129, GSM1258130, GSM1258131, and GSM1258132, from the second dataset. Because of the different backgrounds of the samples, the normalization of these data was performed before enrichment analysis using R packages limma and gplots (Fig. 5a, b). There were 1431 DEGs between those two series, 771 downregulated and 660 upregulated, showed in the heatmap (Fig. 5c). The top 20 KEGG pathways acquired from DAVID (Additional file 3), including focal adhesion, ECM-receptor interaction, and PI3K-Akt signaling pathway, represented the pathways with the highest enrichment scores. In terms of the GO results, chaperone-mediated protein folding and cellular zinc ion homeostasis for BP; and ion channel activity and oxidoreductase activity for MF (Additional file 4) are essential processes of the pancreas. All these results are analogous to the findings of our samples from the transition of “BMSCs” to “islets.”

### PPI network

**Fig. 6:**
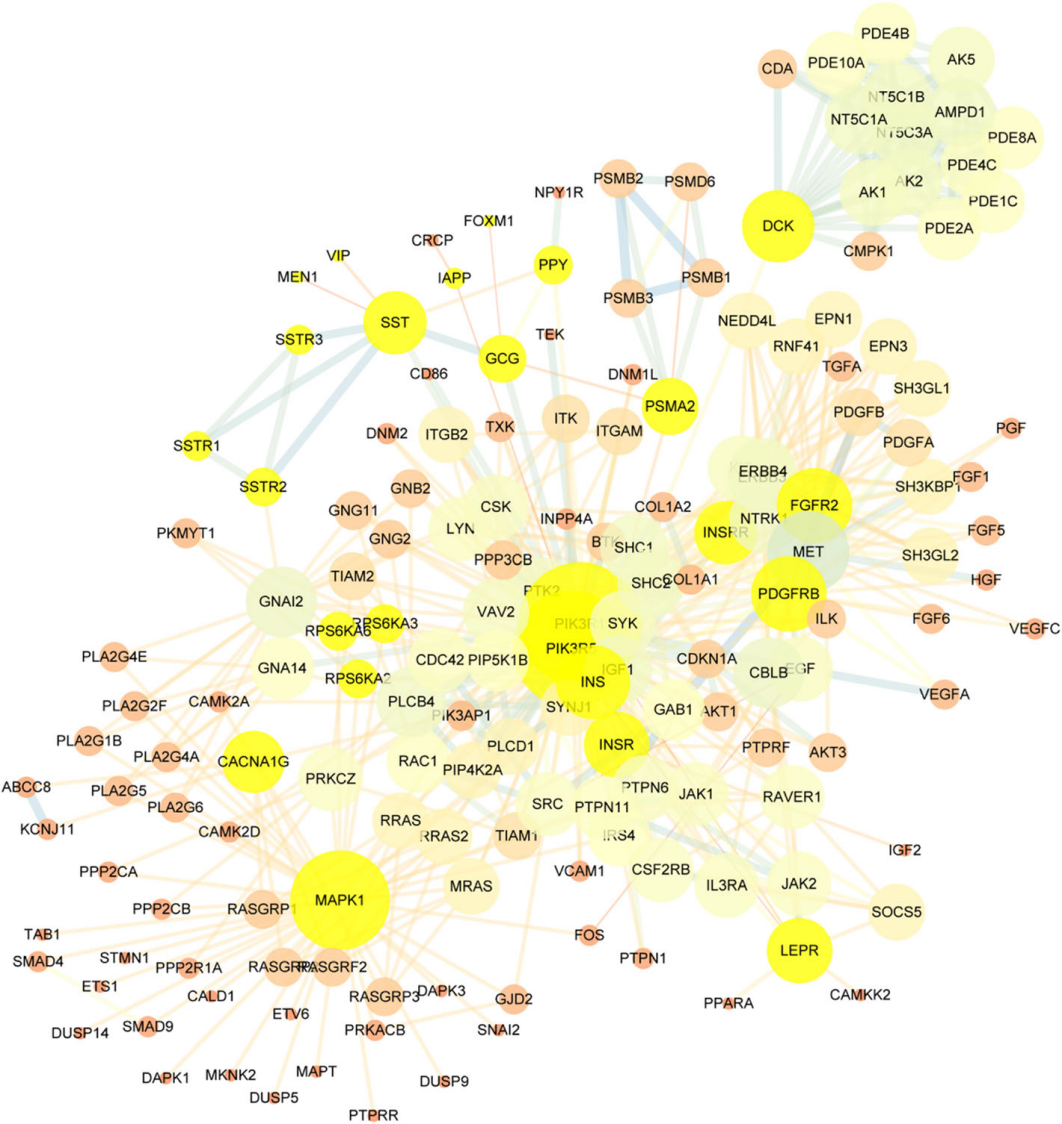
PPI network analysis of known transcripts. The size and color of nodes meant the number of genes related and betweenness centrality, the color and width of edges meant the PPI score of each two genes

The PPI network (Fig. 6) contained 1406 nodes. Insulin, glucagon, and somatostatin represented key roles in this network based on the node size, so we analyzed the PPI network focusing on these three and those who linked to them by the first and second edges. Under these conditions, 168 nodes were shown in this network, and 22 nodes directly interacted with the three key genes.

### Verification and quantification of key genes

**Fig. 7:**
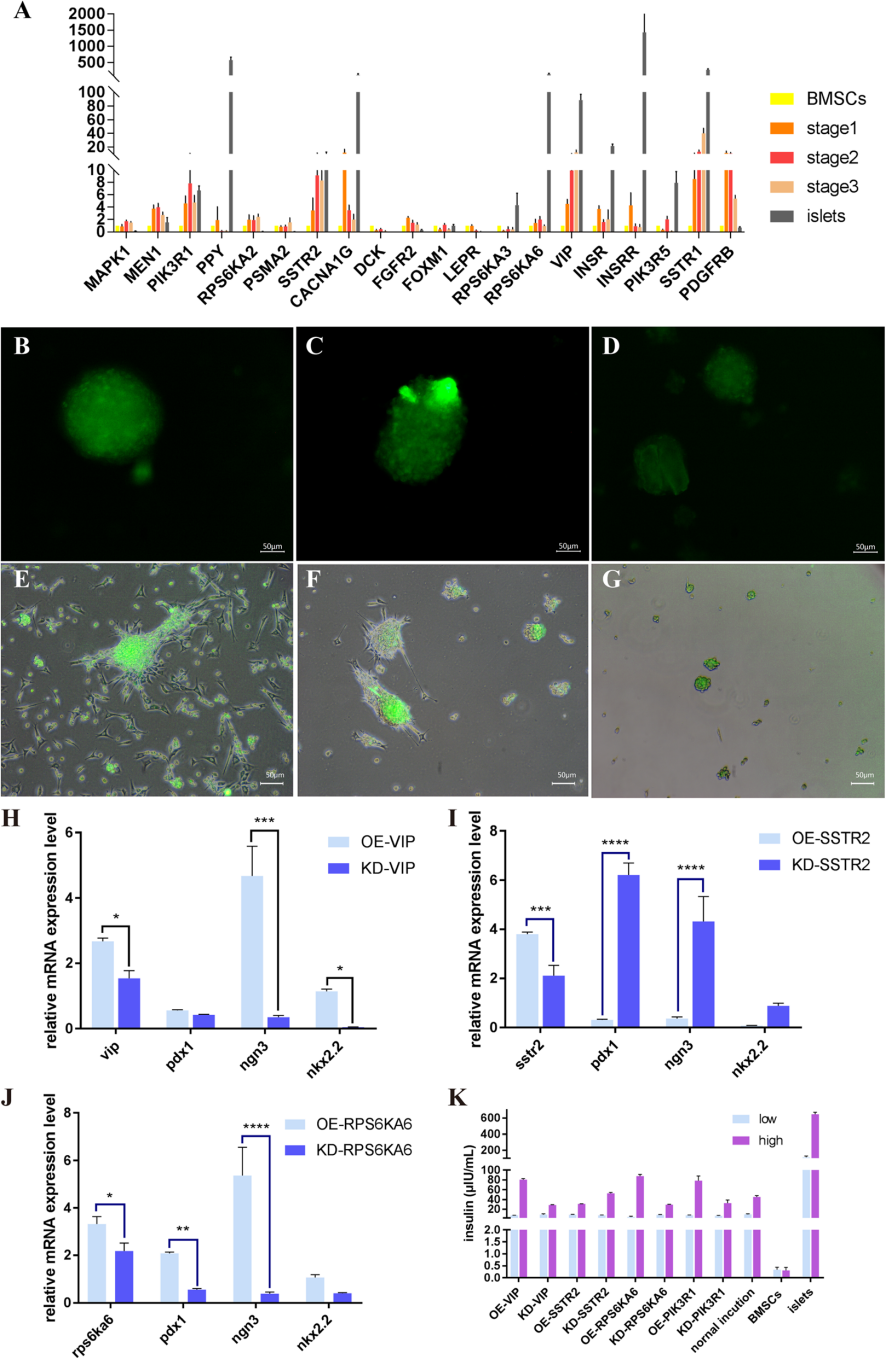
**a** The verification of the 20 key genes by qRT-PCR. **b-d** Overexpression of *VIP, SSTR2* and *RPS6KA6* respectively, 48 h after transfection. **e-g** Knockdown of *VIP, SSTR2* and *RPS6KA6*, the bright fields were merged with fluorescence images. (scale bar = 50 μm) **h** qRT-PCR of OE-*VIP* and KD-*VIP* after induction, “*“means 0.01 < *p* value < 0.05, “**” 0.001 < means p value < 0.01, “***” means 0.0001 < p value < 0.001, “****” means p value < 0.0001. **i** qRT-PCR of OE-*SSTR2* and KD-*SSTR2* after induction. **j** qRT-PCR of OE-*RPS6KA6* and KD-*RPS6KA6* after induction. **k** The GSIS results of overexpression and knockdown of *VIP, SSTR2* and *RPS6KA6* and normal induction groups are control samples

The expression quantity of these 20 genes were verified by qRT-PCR, which was in accordance with the sequencing data (Fig. 7a). Among these genes, *SSTR2*, *RPS6KA6*, and *VIP* were picked out, transfected by lentivirus which were built for overexpression and knockdown of these three genes. It could be found that there were more spheroids in better shape than those who were knockdown (Fig. 7b-g). After the induction procedure, it was showed that the expression level of *PDX1*, *NGN3* and *NKX2.2* were upregulated in the overexpression groups of *VIP* and *RPS6KA6* (Fig. 7h, j), which was contrary for OE-*SSTR2* groups (Fig. 7i). The result gave us a hint that *VIP* and *RPS6KA6* might regulate the expression level of pancreas key genes *PDX1*, *NGN3* and *NKX2.2* through directly interaction or participating the signaling pathway such as MAPK signaling pathway in terms of *RPS6KA6* was a member of it. SSTR2, as a receptor of somatostatin, could have an effect on proliferation and differentiation via lowering cAMP [35]. Meanwhile, the GSIS (Fig. 7k) result was the same outcome.

## Discussion

### Canine BMSCs are advantageous source of pancreatic islet-like clusters

To the best of our knowledge, this is the first study analyzing the transdifferentiation of canine BMSCs according to the timeline of induction. Screening for DEGs between different timepoints was performed, and referring to pancreatic development, we could tell what state the cells were in during the induction, enabling adjustment of the induction procedure to achieve optimal conditions. The reason why we chose canine BMSCs for this study was as follow: (1) They can be easily derived from bone marrow, along with having another advantageous immune escape mechanism compared with other cell types such as ESCs and iPSCs [36–38]; (2) Another advantage is that the usage of and access to these latter two types of cell are often limited by ethical concerns [39]; (3) Canine has always been neglected to be a model animal with the increasing diabetes morbidity of companion dogs, and the same living environment with human can give more help for human diabetes treatment than mouse or any other animals [40, 41]; (4) Canine diabetes is more alike with human type I diabetes, but individual immune variation and environment can make big difference in the development of diabetes [42]. Some abnormal expressed genes involved with human diabetes have been identified in dog diabetes cases [43]. Based on the similar pathology and physiology with human, canine without doubt is an ideal translational disease model for type I diabetes treatment in human [44].

### For the first time induction procedure could be improved based on the transcriptome profiling data

At present, existing methods for induction have low efficiency [45], and no consensus has yet been reached regarding the mechanism of transdifferentiation of BMSCs into insulin producing cells. However, surprisingly, besides our team, no other groups have focused on the timeline-based analysis of the whole transition process of canine BMSCs, and with transcriptome profiling, the data has been uploaded on SRA database (https://www.ncbi.nlm.nih.gov/sra) with accession number PRJNA530799.

The utility of GEO datasets is progressively expanding [46, 47]. In this research, several GEO-derived expression profiles obtained by array were considered. However, when limiting the selection to “canine species”, there were only 174 series selected from the database, which was too few for comparison with the total of 110,121 series of all GEO datasets (as of March 6, 2019). We screened out 71 series belonging to the same platform GPL3738, among which only three series had several samples related to the pancreas and BMSCs. Seven samples were chosen for the following data analysis as a reference and for verification of our profiling data. By using R package and online tool DAVID [48], these data were normalization and differentially expressed genes were extracted out to commit for enrichment analysis (Additional files 3 and 4).

Combined with the sequencing data, we found that all of the transcriptome profiling data and GEO data provided the same findings. The DEGs from each set of data were enriched in pathways such as those related to cell junction, PI3K-Akt signaling pathway, ECM-receptor interaction, Rap1 signaling pathway, cell cycle, and cell adhesion molecules (CAMs). The increased GO term binding activity including protein binding and ion binding reflected certain characteristics of the pancreas, generally related to vigorous secretory activity and calcium exchange [49]. Rap1 and Ras are all the downstream of ERK signaling as well as a part of MAPK signaling pathway [50], which can manipulate the transdifferentiation of BMSCs into IPCs by targeting FOS [51] to finally effect proliferation and differentiation of cells [52]. Cell junction and cell adhesion molecules can form a barrier with selection of small molecules, water and proteins to in and out cells to regulate cell differentiation [53, 54]. Under a nonadherent state during induction, cells exhibit similar protein expression pattern and intercellular junctions with organs in vivo [55], which means that cells could influence other cells via paracrine factors like EGF, VEGF, etc. [56].

PPI network analysis could screen out the genes among DEGs that are directly or indirectly regulated by each other, which could play an indispensable role in guiding future study of important regulatory factors in the process of induction. *VIP, SSTR2* and *RPS6KA6* were emerging from the network. After the overexpression and knockdown, we came to the conclusion that the two of them, *VIP* and *RPS6KA6*, had a positive effect on the induction of BMSCs to form islet-like clusters with relatively high insulin secretion, while the expression of *PDX1*, *NGN3* and *NKX2.2* had similar trend, which meant that these gene might interaction with each other. According to report, *VIP* can modulate BMSCs osteogenesis during bone repair [57], meanwhile, more research confirm that *VIP* can participate in immune activities by targeting ILC3 [58, 59]. Interestingly, *SSTR2* may involve in the rhythmic glucagon and insulin secretion [60]. However, there was little reference available about IPCs differentiation. Furthermore, these genes were participating in the same pathway like MAPK signaling pathway [61], Rap1 signaling pathway and Ras signaling pathway, and it could be a lead for the next-step mechanism research of the induction process to obtain high quality islet-like clusters.

## Conclusion

In this study, we have obtained an overview of the pathways involved in the regulation of BMSC transdifferentiation at different timepoints for the first time, and it makes a progress for learning the mechanism. Simultaneously, *VIP, SSTR2*, and *RPS6KA6* were selected out for their positive potential for the induction, and gain-and-lose function verification of them showed that *VIP,* and *RPS6KA6* could positively regulate *PDX1*, *NGN3* and *NKX2.2*, but *SSTR2* was on the contrary. We supposed that fine-tuning of these genes might contribute to transdifferentiation of BMSCs into IPCs and make an advance to the induction procedure.

## Methods

### Animals

The two Chinese rural dogs used in this experiment were purchased from the Experimental Animal Center of Northwest Agriculture and Forest University (Yangling, China). All animal experiments were carried out in accordance with our institute’s laboratory animal requirements and the Guide for the Care and Use of Laboratory Animals (Ministry of Science and Technology of China, 2006).

### Cultivation and identification of primary canine BMSCs

The BMSCs were extracted from the long bones of two female 3-month-old Chinese rural dogs, after anesthesia by injecting zoletil (0.1 ml/kg, Virbac Group, France). Followed by the experiment, they were committed to euthanasia with 200 mg/min intravenous infusion of propofol till no heartbeat. The bodies were collected by the Experimental Animal Center of Northwest Agriculture and Forest University (Yangling, China) for harmless disposal. For details about the separation of BMSCs, see our previous paper [62]. The cells were identified by flow cytometry and confirmed to undergo three-lineage differentiation the same as when proliferating in dishes [63]. Specifically, the potential for differentiating into the three lineages was assessed by a 7-day induction procedure [64].

### The induction of BMSCs to transform into IPCs

The protocol used in this study involved three stages, while the BMSCs were placed in a suspended state to form spheroids, which mimicked the characteristics of islets in the pancreas. The dishes used in the protocol were all treated with 2-hydroxyethyl methacrylate (Sigma-Aldrich). The medium supplemented with various factors for three stages and the induction procedure have been described in our previous work [5].

Collecting those spheroids with a consistent size, we then performed DTZ staining as described previously and GSIS to determine whether a transition occurred after the induction procedure. The selected clusters were precultured in DMEM low glucose for 2 h to remove the insulin present, washed three times with PBS, and incubated in DMEM low glucose for 30 min. The supernatant was then collected and the clusters were transferred into DMEM high glucose stimulated for 30 min. The released insulin was tested by ELISA using Quantikine® ELISA kit, strictly in accordance with the operating instructions.

### RNA extraction of cell clusters and quality control

Total RNA was fetched by using TRIzol® reagent (Invitrogen, USA), and then we measured the RNA degradation and contamination by DNA gel electrophoresis with 1% agarose gel. The purity of RNA was assessed by using the NanoPhotometer® spectrophotometer (IMPLEN, CA, USA), and Qubit® RNA Assay Kit in a Qubit® 2.0 Fluorometer (Life Technologies, CA, USA) was used to detect the concentration. Finally, RNA integrity (RIN) was determined via RNA Nano 6000 Assay Kit on Bioanalyzer 2100 system (Agilent Technologies, CA, USA).

### Library construction and sequencing processing

We used NEB RNA Library Prep Kit (NEB, USA) for library construction. To preferentially select cDNA fragments of 150–200 bp in length, the library fragments were filtrated using the AMPure XP system (Beckman Coulter, Beverly, USA). The primers used for PCR were Index (X) Primer, and the PCR products were purified using AMPure XP system, as well as the library quality assessment performed on Agilent Bioanalyzer 2100 system.

Cluster generation was performed on a cBot Cluster Generation System using TruSeq PE Cluster Kit v3-cBot-HS (Illumina), in accordance with the manufacturer’s instructions. Subsequently, the sequencing data were processed on an Illumina Hiseq 4000 platform.

### De novo assembly

With raw data going through in-house Perl scripts (Additional file 5), clean data were obtained. Q20, Q30, and GC content were calculated, with Q20 and Q30 representing the percentages of bases whose Phred [Phred = −10log10(e)] equaled or exceeded 20 and 30 relative to all bases. For mapping to the reference genome, the genome and gene model annotation files were downloaded directly from NCBI (https://www.ncbi.nlm.nih.gov/genome/?term=dog) and the annotation release ID was 105. The software HISAT2 v2.0.4 [65] (Additional file 5) was used in this field. Finally, the mapped reads were assembled by StringTie (v1.3.1) [66] (Additional file 5) in a reference-based approach.

### Identification of DEGs

FPKM, which was calculated based on the length and reads, was processed using cuffdiff (v2.1.1) (Additional file 5) and used to define the expression levels of coding genes. The differentially expressed transcripts were screened with an adjusted *P*-value < 0.05. As thresholds for defining significant differences in gene expression between samples, we used |log2FC| > 1 with FDR < 0.05.

### Enrichment analysis of DEGs and PPI analysis

Gene ontology (GO), implemented using the GOseq R package [67], was applied to annotate the functions of DEGs, and they were divided into three biological modules: molecular function (MF), cellular component (CC), and biological process (BP). GO terms with a corrected P-value less than 0.05 were considered to be significantly enriched.

Kyoto Encyclopedia of Genes and Genomes (KEGG) pathway analysis was also adopted to define the functions of the DEGs. KOBAS software were applied to test the statistical significance of enrichment of DEGs in KEGG pathways. The significance formula was the same as that in the GO analysis, and it was presented using the magnitude of –log10 (Q-value) (Q-value was derived from multiple hypothesis testing of the P-value). Meanwhile, RF (rich factor) was defined as the ratio of DEGs relative to the overall annotated transcripts enriched in the same pathway.

PPI analysis of differentially expressed genes was based on the STRING database, which features known and predicted protein–protein interactions. Canine data were included in the database, so we constructed the networks by extracting the target gene list from the database. We visualized the findings using Cytoscape (v3.6.1).

### Lentivirus vectors construction and transfection

The overexpression vector was pCDH-CMV-MCS-CopGFP-T2A-Puro, and complete coding sequences of target genes were synthesized by GeneCreat biological technology Co. (Wuhan, China). The vector used for knockdown was CD513B-U6, and siRNAs were designed by BLOCK-iT™ RNAi Designer (https://rnaidesigner.thermofisher.com/rnaiexpress/). shRNAs derived from siRNAs were synthesized by TsingKe biological technology Co. (Beijing China).

### qRT-PCR

Total RNA was extracted as described above, and cDNA was generated from total RNA. Primers for quantitative real time PCR (qRT-PCR) were designed using Primer Premier 5.0 software and synthesized by TsingKe biological technology Co (Beijing China). qRT-PCR reaction mixture (20 μL) contained SYBR Green qPCR Master Mix (2X) (Thermo Scientific, USA) with three biological replicates. Expression levels of genes were determined by 2^-△△Ct^ based on Ct values.

### Bioinformatic analysis of data from the gene expression omnibus (GEO) database using the DAVID database

The gene expression profiles GSE20113 and GSE52063, chosen from GPL3738, were downloaded from the GEO database; these two series included three normal pancreas samples and four normal bone marrow mesenchymal stem cell samples derived from canine. Because these data were from the same platform, after normalization of the microarray data, we used R (https://www.r-project.org/) to acquire the DEGs, and analyzed these data using the DAVID database (https://david.ncifcrf.gov/).

### Statistical analysis

SPSS 13.0 software was used for analysis of mean, standard deviation and paired T test. Measurement data were presented as mean ± SEM, *p* < 0.05 was statistically significant difference, expressed with (*); 0.01 < p < 0.05 was expressed with (**); *p* < 0.01 for the extremely significant difference, expressed with (***); *p* < 0.001 was expressed with (****). GraphPad Prism 7.0 was used for scientific graphing.

## Supplementary Information

**Additional file 1.** A summary of the quality of output data. “Clean bases” --- The number of reads multiplied by the length of the sequence is converted to G. “Error rate” --- Error rate of base sequencing. “Q20” and “Q30” represented the percentage of bases with Phred value greater than 20 and 30 in the total base. “GC content” represented the percentage of total bases G and C.

**Additional file 2.** A comparison of mapped reads and the reference genome alignment. “Total mapped” --- The number of reads that can be mapped to the genome. “Uniquely mapped” --- The number of reads with a unique matched alignment position on the reference sequence. “Multiple mapped” --- The number of reads with multipul matched alignment positions on the reference sequence. “Reads map to ‘+’” --- The reads that can map to the sense strand of genome. “Reads map to ‘-’” --- The reads that can map to the antisense strand of genome. “Non-splice reads” --- The number of the intact Uniquely mapped reads which map to exons. “Splice reads” --- The number of the segmented Uniquely mapped reads which map to exons. Our samples owned relatively high quality with over 90% mapped clean reads.

**Additional file 3 **KEGG analysis of GEO data using DAVID. It was top 20 signaling pathways showed in this table. “Enrichment Score” --- The overall enrichment score for each term members, the higher, the more enriched. “Count” --- The number of genes involved in individual term. “Banjamini” --- The modified Fisher Exact *P*-Value, the smaller, the more enriched.

**Additional file 4.** Top 20 items of GO analysis of GEO data. “Enrichment Score” --- The overall enrichment score for each term members, the higher, the more enriched. “Count” --- The number of genes involved in individual term. “Banjamini” --- The modified Fisher Exact P-Value, the smaller, the more enriched.

**Additional file 5.** The software and main parameters.

## Data Availability

Authors can confirm that all relevant data are included in the article and its supplementary information files, meanwhile, transcriptome data is uploaded on SRA with accession number: PRJNA530799. The data downloaded from GEO (https://www.ncbi.nlm.nih.gov/geo/) could be accessed via series and samples records GSE20113, GSE52063, GSM502601, GSM502602, GSM502603, GSM1258129, GSM1258130, GSM1258131, and GSM1258132.

## Abbreviations

BMSCs: Bone marrow mesenchymal stem cells
DEGs: Differentially expressed genes
GSIS: Glucose-stimulated insulin secretion
PPI: Protein protein interaction
FPKM: Fragments per kilobase of exon per million fragments mapped
IPCs: Insulin producing cells
PDX1: Pancreatic and duodenal homeobox 1
NGN3: Neurogenin 3
NKX2.2: NK2 homeobox 2
VIP: Vasoactive Intestinal Peptide
SSTR2: Somatostatin Receptor 2
RPS6KA6: Ribosomal Protein S6 Kinase A6
BP: Biological process
MF: Molecular function
CC: Cell component
